# Antiviral Properties of Green‐Synthesized Silver and Zinc Oxide Nanoparticles Against Coronaviruses: A Review

**DOI:** 10.1155/ijm/7207868

**Published:** 2026-06-17

**Authors:** Mohamed E. A. Mostafa, Ahmed E. Ahmed, Mustafa N. Abdulqader, Safaa A. M. Abdel-Karim, Atef S. Elgebaly, Soheir A. A. Hagras

**Affiliations:** ^1^ Department of Anatomy, Faculty of Medicine, University of Tabuk, Tabuk, Saudi Arabia, ut.edu.sa; ^2^ Department of Medical Instruments Engineering, Medical Technical College, Al-Farahidi University, Baghdad, Iraq, uoalfarahidi.edu.iq; ^3^ Department of Medicine, College of Medicine, Ninevah University, Ninevah, Iraq, uoninevah.edu.iq; ^4^ NILES Medical Application Department, National Institute of Laser Science, Cairo University, Cairo, Egypt, cu.edu.eg; ^5^ Department of Microbiology and Immunology, Faculty of Pharmacy, Zagazig University, Zagazig, Egypt, zu.edu.eg; ^6^ College of Medical Laboratory Techniques, Al-Farahidi University, Baghdad, Iraq, uoalfarahidi.edu.iq; ^7^ Pharmacy Department, Alnahda College, Al Munsiyah, Riyadh, Saudi Arabia

**Keywords:** antiviral activity, coronaviruses, green synthesis, silver nanoparticles, zinc oxide nanoparticles

## Abstract

It is acknowledged that COVID‐19 pandemic triggered by SARS‐CoV‐2 outbreak demands the development of efficient antiviral modalities independent of traditional vaccines and antiviral reagents. Metallic nanoparticles, such as silver nanoparticles (Ag‐NPs) and zinc oxide nanoparticles (ZnO‐NPs), have attracted considerable attention as promising antiviral agents due to their distinctive physicochemical properties and universal antimicrobial activity. This article examines the progress made between (2021 and 2025) in the green synthesis and the antiviral applications of Ag‐NPs and ZnO‐NPs against Coronavirus. The environmental friendly synthesis protocols using natural plant juices have been highlighted in this article due to their lower toxicity and greater biocompatibility. Various characterization methods, such as scanning electron microscopy (SEM), transmission electron microscopy (TEM), X‐ray diffraction (XRD), and UV–Vis spectrophotometry, have been used to examine nanoparticle morphology, stability, and surface characteristics. The findings reveald that Ag‐NPs can suppress the viral replication by interacting strongly with S proteins, thereby hindering the cellular entry of the targeted Coronavirus. In contrast, ZnO‐NPs showed that they have dual purposes: direct antiviral effects and immunomodulatory effects that involve cytokine regulation. The antiviral properties and the limitations of the individual nanoparticles are significantly modified by hybrid nanocomposites. In closing, the significance of using green‐synthesized Ag‐NPs and ZnO‐NPs as antiviral materials is tremendous and warrants further exploration for antiviral applications. Nonetheless, there are still some unaddressed areas concerning the standardization of synthesis techniques and the validation of prolonged safety. Finally, it has been proven that there is a need for collaboration between nanotechnology and green technology in the development of environmentally benign, and universal antiviral medications against any future coronavirus pandemics.

## 1. Introduction

All countries have been prompted to make intensive and persistent efforts to develop effective diagnostic, preventive, and therapeutic strategies to combat coronavirus pandemic which causing a severe acute respiratory syndrome 19 (SARS‐CoV‐19), the causative agent of the Coronavirus disease 2019 (COVID‐19) pandemic, emerged, which is considered an unprecedented global health challenge [1]. COVID‐19 is a massive viral threat with severe health consequences [2]. Its symptoms range widely, from mild respiratory problems to serious complications affecting multiple organs [3]. The virus has rapid global transmission, overwhelms hospitals, and causes significant socioeconomic disruption [4]. Given the destructive nature of the pandemic, minimizing its severity is critically important, which requires the rapid development of essential vaccines. There is a need for broad‐spectrum antivirals and alternative, integrated therapeutic approaches to keep pace with the ongoing evolution of viral mutations [5]. Owing to the unique physical and chemical properties of nanomaterials, nanotechnology has recently emerged as a promising prospect for combating viral infections [6]. These properties include a high surface area‐to‐volume ratio, tunable volume, and the ability to interact with biological systems at the molecular level [7].

Recently, especially in the medical field, researchers have focused on nanoparticles, including two types of engineered metal microparticles: silver (Ag) and zinc oxide (ZnO) [8]. This interest stems from the well‐documented ability of nanomaterials to combat a wide range of microbes and viruses. These nanoparticles possess broad‐spectrum antimicrobial properties, and their activity has been clearly demonstrated against a wide range of viruses, including influenza virus, herpes simplex virus, and, more recently, novel coronavirus (SARS‐CoV‐2) [9]. These particles demonstrate a superior ability to inhibit viral growth through multiple diverse mechanisms [10]. They directly affect the virus, preventing it from attaching and entering the body cells by binding to its surface proteins, and they generate molecules capable of destroying it [11]. Reactive oxygen species (ROS) play a major role in inducing oxidative damage to viral structures. A comprehensive understanding of the latest developments in nanotechnology is essential, as it enables the integration of Ag and zinc (Zn) into hybrid nanocomposites or composite materials [12]. This development has increased the efficiency of virus control, potentially reducing concerns associated with the use of single‐component nanoparticles, such as toxicity issues or the emergence of specific viral resistance mechanisms [13].

The main objective of this review is to systematically analyze and synthesize the scientific literature published over the past 5 years (2021–2025) on the biological activity of silver nanoparticles (Ag‐NPs) and zinc nanoparticles (ZnO‐NPs) and their composites against coronaviruses, with particular emphasis on the SARS‐CoV‐2. The integration of plant knowledge with nanotechnology offers broad opportunities to develop hybrids with synergistic biological activities. The advantages of green synthesis go beyond the environmental compatibility of these compounds to include functional applications of therapeutic plant compounds, as well as scalability and economic feasibility [2]. However, the challenges in standardizing protocols, ensuring reproducibility, and elucidating the precise mechanisms of nanoparticle formation and effectiveness remain. This review will critically assess the main conclusions about their modes of action, the manner in which physicochemical characteristics (such as size, shape, and surface functionalization) determine their antiviral potential, and whether they can be used in a variety of settings, such as air filtration, surface coatings, and possible therapeutic drug delivery systems.

This study is also aimed to identifying the current research gaps and the future approaches regarding its biocompatibility, in vivo efficacy, and potential for clinical translation as a potent, broad‐spectrum antiviral intervention against current and future coronavirus threats.

## 2. COVID‐19

COVID‐19, also known as SARS‐CoV‐2 disease, is an infectious illness caused by the SARS‐CoV‐2 virus. At the beginning of 2020, the infection spread rapidly worldwide, leading to the declaration of a global pandemic [3]. The signs of the disease vary from person to person, but common signs include fever, tiredness, coughing, breathing difficulties, and the loss of both smell and taste [4]. These symptoms can persist from 1 day to 2 weeks after exposure to the virus [14, 15]. However, it take between a day and 2 weeks for the first symptom to appear after exposure to the virus. One in three, however, shows no signs of the virus. The vast majority of those who do fall ill, approximately 81%, develop only mild to moderate symptoms such as pneumonia. Approximately 14% experience worsening problems, such as shortness of breath, low oxygen levels, or damage to more than half of the lung, as observed on imaging. Only a small subset, approximately 5%, develop severe forms of the disease characterized by acute respiratory failure (i.e., inability to sustain blood oxygen levels) accompanied by profound circulatory collapse or multiorgan failure [5]. Elderly individuals are more likely to develop severe symptoms following infection by COVID‐19. In some instances, complications can be fatal. Others are stuck with long‐term health issues, commonly referred to as “long COVID,” that can linger for months or years after resolution of the acute infection. There is also evidence suggesting that the virus can harm specific organs, and long‐term studies are underway to examine these lingering consequences [6].

## 3. COVID‐19 Transmission

COVID‐19 can spread when a person breathes in infectious particles or when these particles come in contact with the eyes, nose, or mouth [5]. The likelihood of infection increases when people are close to one another, but tiny virus‐carrying particles can linger in the air and travel farther distances, particularly in indoor spaces [6]. COVID‐19 can also spread when an individual touches their eyes, nose, or mouth after coming in contact with surfaces or objects contaminated by the virus [4]. An infected person may remain capable of transmitting the disease for up to 20 days, even if they never show obvious symptoms [4].

## 4. Diagnosis of COVID‐19

Several methods are used to diagnose COVID‐19 by detecting the virus′s genetic material. The most common methods include real‐time reverse transcription polymerase chain reaction (RT‐PCR), transcription‐based amplification techniques, and reverse transcription loop‐mediated isothermal amplification (RT‐LAMP). Typically, a sample is collected via a nasopharyngeal swab [7]. Several COVID‐19 vaccines have been authorized and distributed across different countries, and many of these nations have launched large‐scale vaccination programs [8]. Other protective measures include maintaining physical or social distancing, quarantining when necessary, ensuring good ventilation in indoor areas, wearing masks or face coverings in public, covering the mouth and nose when coughing or sneezing, washing hands regularly, and avoiding touching the face with unwashed hands [9]. Although certain medications have been developed to target the virus and reduce its activity, the main approach to treatment continues to focus on managing symptoms [10]. This is accomplished through supportive care, patient isolation, and, when necessary, the use of experimental methods [11].

## 5. Nanoparticle Synthesis

Many studies have recently focused on the synthesis of metallic nanoparticles. They appeal to scientists because of their distinctive properties, which make them useful for everything from medicine and agriculture to environmental cleanup. Two types that have received attention are Ag‐ and ZnO‐NPs [12]. They are highly effective at battling microbes, acting as antioxidants, and accelerating chemical reactions. Making these particles has been problematic in the past [13]. This old technology is often based on toxic chemicals, requires a ton of energy, and is rather complex. This has prompted many questions about how safe and green they are. Owing to these problems, there has been a migration toward a more sustainable process [16]. They have turned from plants, building nanoparticles from compounds derived from them. The method of “green synthesis” represents a significant advance toward sustainable and eco‐friendly methods [12]. The novelty of using plants to produce nanoparticles lies in the fact that this process leverages several strategic advantages. Taking advantage of naturally occurring chemicals in plants is a smart route. Compounds such as flavonoids, alkaloids, and proteins, which are already synthesized by plants, can play dual roles: they reduce metal salts to nanoparticles and act as stabilizing agents that interact with them [13]. Notably, this entire sequence is also gentle on the planet. It tends to work at room temperature and eliminates the need for hazardous chemicals. Additionally, plant extracts are potentially quite efficient for nanoparticle synthesis, as they contain phytochemicals that are pre‐stored [16]. Compounds such as flavonoids and proteins play essential dual roles by reducing metal ions to NPs through their functional groups and stabilizing them. This is an inherently green process; it generally takes place at room temperature without any hazardous chemicals [17]. Most importantly, the result is very tunable. The final shape, size distribution, and surface properties of these nanoparticles are primarily controlled by the plant source, extraction process, and synthesis parameters [14]. The pharmaceutical advantages of biosynthesized ZnO‐NPs and Ag‐NPs, possibly via plant extracts, include, but are not limited to, their functional performance, biological compatibility, and reduced cytotoxicity [15]. Thus, green‐synthesized particles are highly effective against various Gram‐positive and Gram‐negative bacteria, fungi, and a few viruses [18]. According to numerous studies and the use of extracts from specific medicinal plants, such as *Ocimum sanctum* (holy basil), *Camellia sinensis* (green tea), *Azadirachta indica* (neem), and *Moringa oleifera*, Ag‐NPs and ZnO‐NPs have been successfully synthesized [19]. They are extracted via ethanolic or aqueous extraction, after which they are combined with solutions of mineral salts, for example, Ag nitrate (Ag‐NO_3_) or Zn acetate (Zn[CH_3_COO]_2_) [20]. This change in the color is a clear evidence of the formation of nanoparticles [21]. By means of the advanced techniques, such as scanning electron microscopy (SEM), Fourier transform infrared (FTIR), ultraviolet–visible spectroscopy, transmission electron microscopy (TEM), and X‐ray diffraction (XRD) analyses, we have determined the properties of these materials [22].

## 6. Synthesis by Green Chemistry

A pervious study [23] demonstrated that the green production of Ag‐NPs using medicinal plants is characterized by their environmental safety, lack of reliance on harmful components, and the simplicity and high efficiency in producing effective nanoparticles. In addition, plants generate these nanoparticles by absorbing, utilizing, accumulating, and employing various nutrients in multiple ways [24]. Moreover, a previous study revealed important details about Ag‐NPs manufactured by plants [25]. On the other hand, the synthesis of metallic nanoparticles via plants has significant advantages over chemical, physical, or microbiological methods, as it is a rapid, reproducible, environmentally friendly, economical, and widely applicable procedure [26].

## 7. Synthesis of ZnO‐NPs

Several components, including *Aspergillus niger* [27], *Hibiscus rosa-sinensis*, and Cassia flower [28], can be utilized to finish the synthesis process. The amount and concentration of Zn nitrate utilized in the production of ZnO‐NPs have been reported to be reduced by using plant extracts [29]. The type of plant or the source of the plant extract, such as European olive oil, used in the production of Zn nanoparticle sheets, influences the size of the nanoparticles produced from plant extracts [30]. The outcomes were promising. The size, for instance, varied between 18 and 30 nm. ZnO‐NPs have been found to have antibacterial properties in recent research [31]. Moreover, the typical size of the nanoparticles ranges from 25 to 40 nm when *Aloe barbadensis* and *Ocymum tenofiflorum* are used as reducing agents for the green production of ZnO‐NPs [32]. Furthermore, ZnO‐NPs extracted from the leaves of passionflower [33], *Passiflora caerulea*, and *Camellia sinensis* have shown antibacterial activity, particularly against *Klebsiella pneumoniae* and *Pseudomonas aeruginosa*[34], which means that the production of ZnO‐NPs by means of the medicinal plant extracts may be beneficial to the medical sector [34].

**Figure 1:**
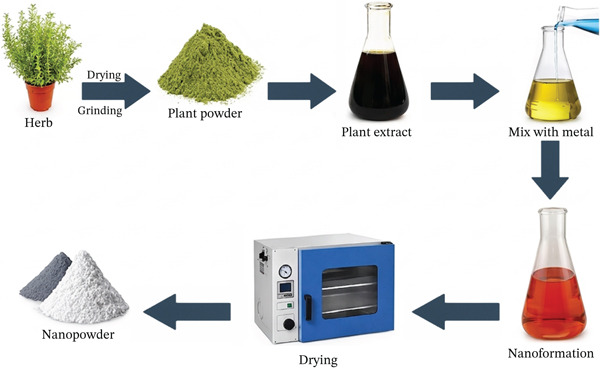
Schematic representation of the green synthesis of plant‐mediated metal nanoparticles. Plant material is dried, ground, and extracted to obtain bioactive compounds that reduce and stabilize metal ions during nanoparticle formation. The resulting nanocolloid is subsequently dried to produce nanopowder via an eco‐friendly and sustainable approach.

ZnO‐NPs have a superior ability to penetrate both the surface and the core of bacteria when reduced to the nanometer scale, depending on the entry point, and exhibit effective mechanisms for killing the bacteria [35]. Intrinsic properties play a significant role in shaping the size, composition, and crystallinity of metallic nanoparticles, such as ZnO‐NPs [36]. Properties, such as the chemical and optical characteristics of each nanocomposite, change once materials are reduced to a nanocomposite form. ZnO‐NPs can easily enter cell components and interact with their vital components. Furthermore, a significant degree of surface interaction arises from increasing the surface‐to‐volume ratio of nanomaterials. As a result, nanomaterials are crucial in many fields, including medical and applied sciences [37], as shown in Figure 1.

**Figure 2:**
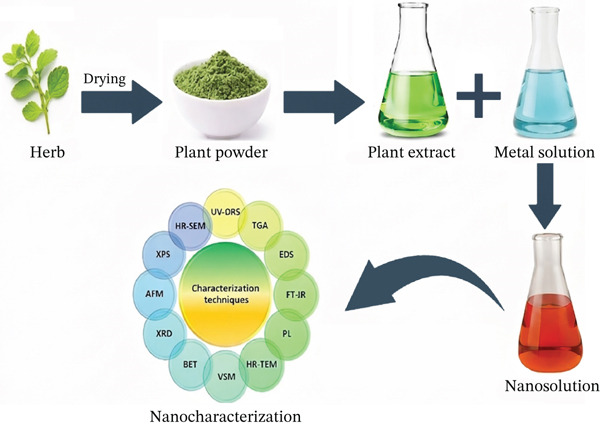
Schematic overview of the green synthesis of metal nanoparticles using plant extracts. Plant material is dried, ground, and extracted to obtain bioactive compounds, which are then mixed with a metal precursor to form a nanosolution. The synthesized nanoparticles are subsequently characterized using various physicochemical techniques.

To better understand the physical and chemical behavior of Ag‐ and ZnO‐NPs and to enhance their use in biomedical and environmental applications, accurate characterization is crucial [38]. These nanoparticles may be spherical, rod‐shaped, or irregularly shaped. These nanoparticles vary in size, typically 10–100 nm [39]. TEM and SEM are widely used to image the particle shape, surface texture, and size distribution at the nanoscale [22]. Dynamic light scattering (DLS) measurements provide complementary information on the hydrodynamic diameter and colloidal stability in suspensions [40]. FTIR spectroscopy is used to obtain information about the biomolecules that are responsible for capping and stabilizing the nanoparticles, particularly in green synthesis procedures, as well as to establish the functional groups on the surface of the nanoparticles [41]. XRD analysis was carried out to confirm the crystalline morphology of the nanoparticles, and characteristic diffraction peaks were observed that can be attributed to face‐centered cubic (fcc) Ag ZnO structures or hexagonal wurtzite ZnO structures [42]. To observe the stages of nanoparticle formation in a quick, nondestructive, and spectral manner, a UV–Vis spectrometer can be used because ZnO‐NPs are absorb in the UV region owing to their wide bandgap, and Ag‐NPs typically have a surface plasmon resonance (SPR) peak at 400–450 nm. Zeta potential analysis is also important for forecasting stability against dispersion and assessing long‐term surface charge [43]. Furthermore, thermogravimetric analysis (TGA) provides thermal stability data, organic content information, and differential scanning calorimetry (DSC) data, which are essential for tailoring performance in antimicrobial, catalytic, and drug‐delivery applications [44]. Therefore, the following technologies provide a comprehensive understanding of the properties of nanoparticles, and the integration of the multiple characterization tools ensures the reproducibility, the safety, and the functional effectiveness of nanomaterials in real‐world environments [45] as shown in Figure 2.

## 8. Antiviral Activity of the Ag‐NPs

Acquired immunodeficiency virus (e.g, HIV) is a well‐known virus [46]. Some researchers have investigated how Ag‐NPs prevent HIV infection and speculated that these nanoparticles might have an anti‐HIV effect by contributing to the early phases of the virus replication cycle, most likely as dependable agents or inhibitors of viral entry [47]. The antiviral potential of Ag‐NPs has been demonstrated by their ability to prevent CD4‐Mediated virion binding, fusion, and infectivity, as well as by the gp120 protein′s ability to bind and interact with target cell membrane receptors [48]. The silver nanoparticle binding method to the Gp120 protein makes it a reliable and potent agent against viruses (laboratory and resistant strains) [49]. Additionally, it inhibits the progression of HIV to the next phase of its life cycle. These characteristics suggest that Ag‐NPs can be used to prevent the transmission of HIV‐1 because they have broad‐spectrum antiviral activity and are resistant to the resistance [50].

Silver and gold nanoparticles coated with mercaptoethanesulfonate have also been tested for their strong antiviral activity against herpes simplex virus Type 1 (HSV‐1) [51]. The interaction between herpes simplex virus C glycoprotein and heparin sulfate is crucial for the virus to adhere to the cell surface [52]. The interaction between herpes simplex virus C glycoprotein and heparin sulfate is crucial for the virus to adhere to the cell surface. By interfering with viral adhesion, gold‐coated nanoparticles decrease the persistence of the viral infection as well as the initial entry and multimediated dissemination of the virus from cell to cell, according to additional mechanistic studies [53].

When fungal cell filtrates were treated with aqueous Ag nitrate solution, a significant reduction in the replication efficiency of certain viruses, such as HSV‐1 and HPIV‐3, was observed, with only a limited effect on HSV‐2 [54]. Ag‐NPs prepared from *F. oxysporum* have the greatest potential to inhibit RDV (i.e., HSV‐1 and HPIV‐3), although their size is smaller than that of other nanoparticles, which makes them also toxic at higher concentrations [55]. Due to their antiviral effects, interactions between Ag nanocomposites and viruses, including interactions with pathogens at the cellular level, can also diminish viral infectivity in cells, which is determined by nanoparticle size and zeta potential. Smaller nanoparticles were more effective in decreasing the infectivity of the viruses under study [56].

## 9. The Antiviral Impact of Ag‐NPs on SARS‐CoV‐2

Ag‐NPs have attracted an increasing attention over the last 5 years due to their broad antimicrobial effects, particularly their significant antiviral activity against the SARS‐CoV‐2 [57]. This is mainly due to their capacity to directly interact with the viral architecture and the host cell membranes, taking advantage of their nanometric size and high surface reactivity [58]. These interactions impair processes of the viral replication and mechanisms of cellular ingress. Recent laboratory experiments have also shown that Ag‐NPs can decrease the infectivity and replication capacity of SARS‐CoV‐2 [59]. This reduction is mediated by interactions and binding to the viral spike protein, thereby impeding its attachment to ACE2 receptors on human cells [60]. In addition, it has been suggested that Ag‐NPs can cause structural deformation of the viral surface and consequent irreversible inactivation of the virus [61]. Medical applications of Ag‐NPs have been explored in vivo. A 2022 study reported that Ag‐NPs in nasal sprays and surface disinfectants significantly reduced infection transmission among frontline healthcare workers. These results show that Ag‐NPs might be used as another protective armamentarium for COVID‐19, especially in high‐risk clinical settings [61].

**Figure 3:**
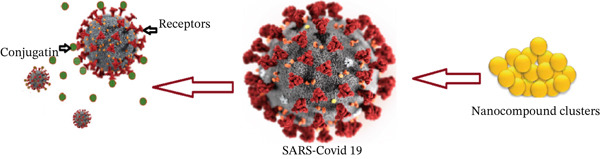
Schematic illustration of the interaction between nano compound clusters and SARS‐CoV‐19. Nanoparticles bind to viral surface receptors through functionalized conjugates, potentially inhibiting viral attachment and entry. This highlights the prospective antiviral mechanism of nanomaterials against COVID‐19.

Importantly, the well‐designed structure of Ag‐NPs, in addition to their low cytotoxicity and good biocompatibility, makes them potential candidates for use in topical formulations, protective equipment, and air protection [62]. However, long‐term safety and the development of uniform clinical protocols are topics requiring continued investigation. In summary, Ag‐NPs may represent an attractive approach for addressing the COVID‐19 pandemic. Owing to their unique physicochemical properties, they offer a multifaceted approach to viral blockade that is potentially applicable to both prophylaxis and treatment [63], as shown in Figure 3.

## 10. Efficacy of ZnO‐NPs Against SARS‐CoV‐19

ZnO‐NPs have recently attracted considerable interest due to their antiviral potential, especially against SARS‐CoV‐2 [64]. Owing to their nanoscale size and pronounced surface reactivity, these particles can interact with the viral structures and host cell components, thereby interfering with the progression of the infection cycle [65]. In addition to their direct antiviral activity, ZnO‐NPs have demonstrated a notable capacity to modulate immune responses. These compounds can promote the production of antiviral cytokines while simultaneously inhibiting inflammatory markers, which may contribute to treating the cytokine storm in patients with severe COVID‐19 [66]. Therefore, ZnO‐NPs serve as an integrated weapon in the dual approach to pandemic healthcare, including both viral blockade and immune regulation. In line with these findings, ZnO‐NP‐based sprays have been developed for surface sterilization and to improve the performance of personal protective equipment, as a practical realization of these findings [67]. The experimental results confirmed a decrease in viral loads on treated surfaces and an increase in biosafety standards within the healthy environment. Despite the relatively low cytotoxicity of ZnO‐NPs at controlled dosages [68], still there is an urgent need for more studies to standardize preparation methods, develop delivery strategies, and test potential side effects in long‐term clinical use [69], as revealed in Figure 3.

## 11. Antimicrobial Activity of Ag‐NPs

Owing to their broad‐spectrum activity against various pathogenic microorganisms (*Escherichia coli* [*E. coli*], *Staphylococcus aureus* [*S. aureus*], and *K. pneumoniae*) in addition to their combined interaction with antibiotics, Ag‐NPs are considered gold surface materials for health sciences and nanotechnology studies [70]. These NPs can be incorporated into a wide range of biological matrices, such as catheters and surgical dressings, to prevent bacterial adherence, thereby reducing infections across multiple surfaces [71]. In addition, Ag‐NPs are used in diverse applications, such as burn healing, the elimination of microorganisms from textiles, and the decontamination of water [72]. Furthermore, studies have shown that coating Ag nano compounds on graphene oxide nanoparticles results in a strong antibacterial and antibiofilm activity. Moreover, some Ag‐NPs have demonstrated excellent growth inhibition of *P. aeruginosa* at minimum inhibitory concentrations (MICs) ranging from 2.5–5.0 g/mL, completely preventing bacterial attachment to stainless steel surfaces [73]. The incorporation of Ag nano compounds into nanofiber scaffolds has also been recently reported to prepare novel materials for use as antibacterial the wound dressings [74]. These advances pave the way for new applications in robust biomedical products focused on wound care and infection.

For example, Ag‐NPs have been tested against various *Candida* and *Aspergillus* species, revealing interesting results regarding their ability to inhibit growth [75]. Furthermore, Ag, copper (Cu), and Zn‐based nanoparticles are becoming increasingly important for their anti‐fungal activity, as they also exhibit antibacterial activity, thereby increasing their global medical and ecological value [76]. It has been suggested that the antifungal effects of Ag‐NPs could be due to the impact of Ag‐NPs on intracellular biological activities, which may cause fungal cell death through direct effects (mainly penetration through the cell wall) [77]. These nanoparticles can release Ag ions, which are believed to damage genetic material (e.g., DNA) and form unstable protein structures, thereby causing leakage of cellular content through the formation of radical oxygen species (ROS) [78]. Furthermore, a previous studies reported that the indirect role of FA in antifungal activity mediated by Ag‐NPs in plant‐pathogenic fungi is significantly related to the concentration and structural properties of the NPs. Notably, fungal toxins are usually not as toxic as bacterial toxins are [79].

## 12. Antimicrobial Activity of ZnO‐NPs

**Figure 4:**
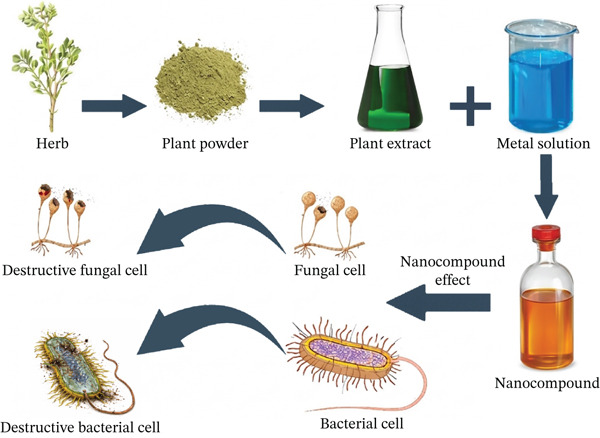
Schematic illustrates the green synthesis of plant‐based nanocompounds. Herbs are processed into powder and then extracted, followed by mixing with a metal solution to form nanoparticles. These nanocompounds demonstrate strong antimicrobial activity, leading to the destruction of fungal and bacterial cells.

Research addressing the antibacterial properties of the biologically synthesized Ag‐NPs revealed that the studied bacterial strains were more susceptible to these nanostructures than Fungi *A. versicolor* [9]. Furthermore, researchers have emphasized the ability of some fungi to excrete low‐molecular‐weight organic acids, which reflect complex interactions between the fungal cells and NPs, but further studies are needed for abetter understanding. Furthermore, it has been reported that ZnO‐NPs (30 nm) have a pronounced bactericidal effect by directly interacting with the phospholipid bilayer to destroy the plasma membrane [80]. The addition of radical scavengers, mannitol, vitamin E, and glutathione to ZnO‐NPs reduced the bactericidal activity, confirming that ROS production plays a key role in their antibacterial action [81]. A previous study [31] investigated the effects of ZnO‐NPs on Vibrio cholerae for the design of a nanomedicine against cholera. The results revealed that the El Tor biotype (N16961) was more resistant to ZnO‐NPs, and the mechanism was increased ROS offloading. The integrity of the bacterial cellular membrane has also been shown to be lost; bacteria have become permeable and undergone severe morphological changes [31]. In addition, ZnO‐NPs were shown to exhibit antibacterial activity in a mouse model of cholera toxin (CT) infection. Other results indicated that these nanoparticles disrupted the secondary structure of the CT and interfered with its binding to the GM1 ganglioside receptor, thereby reducing its effectiveness [82] as revealed in Figure 4.

## 13. Biological Activities and Mechanism of Action

### 13.1. Antimicrobial Activity

This is one of the most investigated properties of nanoparticles. Ag‐ and ZnO‐NPs have shown promising antimicrobial activity against bacteria, fungi, and viruses, including drug‐resistant strains [83].

### 13.2. Mechanism of Action

The antimicrobial activity of these nanoparticles can be attributed to multiple routes. The production of ROS, which leads to an oxidative stress and cellular injury, is one of these [84]. Furthermore, they can directly damage microbial cell membranes and release metal ions that suppress enzymatic activity, interfere with DNA stability or integrity, disrupt numerous cellular physiological activities, and lead to the microbial death [84].

### 13.3. Antioxidant and Anti‐Inflammatory Effects

Several naturally occurring metal and metal oxide nanoparticles have shown well‐defined antioxidant properties in the scavenging of free radicals [85]. This activity is frequently associated with their anti‐inflammatory potential, suggesting a possible role in the management of diseases associated with oxidative stress [86].

### 13.4. Antidiabetic Potential

In recent studies, some nanoparticles have been reported to enhance glucose uptake by the cells and inhibit key enzymes, such as *α*‐amylase [87]. This leads to a significant hypoglycemic effect in diabetic animal models and to the regeneration of pancreatic and hepatic tissues [88].

### 13.5. Anticancer Properties

Due to their effects, nanomaterials are considered accurate and effective approaches for cancer treatment and can be modified to selectively target either tumor cells or the tumor microenvironment [89]. These cells can directly enter cancer cells and subsequently cause the death of the host cell through several biochemical pathways, such as apoptosis, necrosis, and autophagy [90]. In addition, it can specifically target CSCs, thereby reducing the risk of tumor relapses or metastasis [90].

## 14. Applications in Therapeutics and Diagnostics

In addition to their intrinsic bioactivities, nanoparticles have become indispensable tools in drug delivery and bioimaging

### 14.1. Targeted Drug Delivery

Targeted drug delivery nanoparticles serve as excellent vehicle carriers for drugs and can encapsulate various therapeutic agents, thereby increasing their solubility, bioavailability, and stability in the body. Crucially, they may also be functionalized to target specific pathological tissues (e.g., tumors) while preserving healthy cells. This allows unprecedentedly accurate freedom in drug release [91].

### 14.2. Bioimaging and Diagnostics

Owing to their specific optical, magnetic, and electronic properties, nanoparticles can serve as efficient contrast agents for several imaging technologies, such as magnetic resonance imaging (MRI) and fluorescence imaging [92]. High‐contrast imaging materials, such as quantum dots and gold nanoparticles, are used to visualize cellular structures, biomarkers, and pathogenic organisms [93].

## 15. Harmful Impacts of Nanoparticles on the Human Body

Although green‐synthesized Ag‐ and ZnO‐NPs are biocompatible, they may cause unexpected biological reactions. Their small size allows cellular penetration, which can cause membrane and DNA damage by producing excessive ROS and oxidative stress. Exposure has been linked to alterations in immune signaling pathways, which may lead to immunological imbalance or inflammatory responses. Changes in cellular metabolism and tissue damage may result from accumulation in organs such as the liver, kidneys, and lungs. Prior to biological or therapeutic application, thorough toxicological screening and strict dose control are therefore crucial [94].

## 16. Bioavailability and Clearance of Nanoparticles

The permeation of green‐synthesized Ag‐ and ZnO‐NPs across the biological barriers is determined by their dimensions, surface chemistry, and mode of exposure, all of which impact their bioavailability. After systemic dispersion, they undergo partial biotransformation, which influences cellular metabolic processing by forming a protein corona and releasing ions. The renal and hepatobiliary pathways serve as the principal mechanisms of elimination, but diminished clearance may facilitate temporary accumulation in specific tissues [95].

## 17. Comparative Performance of Nanoparticle and Traditional Antiviral Medications

Nanoparticle‐based antivirals improve drug distribution, stability, and tailored efficacy compared with traditional antiviral treatments. They can operate via several methods, such as inhibiting viral entry and modulating the immune responses, potentially reducing the required treatment dosages. Nevertheless, conventional antivirals are better clinically validated, with the distinct safety and pharmacokinetic profiles [96].

## 18. Conclusion

Investigation of Ag‐ and ZnO‐NPs has demonstrated their high potential for use as next‐generation antiviral agents against SARS‐CoV‐2 and similar viruses. Because these materials have nanoscale size, surface reactivity, and modulatory effects on host responses, this multilateral defense unit exceeds the capabilities of classical therapeutics. Green synthesis methods also increase their biocompatibility and sustainability, providing opportunities for a large‐scale biomedical use. Findings have demonstrated their potential role in blocking the viral entry, hampering replication, and promoting protection under the clinical and the environmental conditions. However, problems with the standardization of the synthesis procedure, the reproducibility, and the long‐term safety still are needed to be solved. Closing these gaps will be necessary to translate the laboratory successes into real‐world interventions. In the end, Ag‐ and Zn‐based nanomaterials remain at the national forefront of the antiviral innovation, providing an adaptable platform for the pandemic preparedness and further therapeutic development.

**Nomenclature**
Ag‐NPs: silver nanoparticles
COVID‐19: Coronavirus disease 2019
FTIR: Fourier transform infrared spectroscopy
*GP120*
: *glycoprotein120*
HIV: human immunodeficiency virus
HSV: herpes simplex virus
nm: nanometers
NPs: nanoparticles
ROS: reactive oxygen species
*SEM*
: *scanning electron microscopy*
SPR: surface plasmon resonance
TEM: transmission electron microscopy
UV–Vis: ultraviolet–visible
*XRD*
: x‐ray diffraction analysis
XPS: x‐ray photoelectron spectroscopy
ZnO‐NPs: zinc oxide nanoparticles

## Author Contributions

All the authors contributed equally to the study conception, literature review, drafting, and critical revision of the manuscript.

## Funding

No funding was received for this manuscript.

## Disclosure

All the authors read and approved the final version.

## Ethics Statement

The authors have nothing to report.

## Conflicts of Interest

The authors declare no conflicts of interest.

## Data Availability

The data that support the findings of this study are available from the corresponding author upon reasonable request.
